# Causes of higher symptomatic airway load in patients with chronic rhinosinusitis

**DOI:** 10.1186/s12901-017-0048-6

**Published:** 2017-12-29

**Authors:** Øystein Eskeland, Kjell Arild Danielsen, Fredrik Dahl, Katrin Fridrich, Vivian Cecilie Orszagh, Gregor Bachmann-Harildstad, Espen Burum-Auensen

**Affiliations:** 1 Department of Otorhinolaryngology, Akershus University Hospital, Lørenskog, Norway; 2 Drøbak Ear Nose Throat, Drøbak, Norway; 3 University of Oslo, Institute of Clinical Medicine, Campus Ahus, Lørenskog, Norway; 4 Department of Otorhinolaryngology, Østfold Regional Hospital, Kalnes, Norway; 5 Health Services Research Unit, Akershus University Hospital , Oslo, Norway; 6 Department of Pathology, Akershus University Hospital, Lørenskog, Norway; 7 Biogen, Oslo, Norway

**Keywords:** Chronic rhinosinusitis, Nasal polyps, Endoscopic sinus surgery, SNOT-20, Vas, Lund-Mackey CT score, Biofilm

## Abstract

**Background:**

Chronic rhinosinusitis display a variety of different phenotypes. The symptoms of disease are characterised by various signs and symptoms such as nasal congestion, nasal discharge, pressure sensation in the face and reduced or complete loss of smell.

In a patient population undergoing functional endoscopic sinonasal surgery (FESS) for chronic rhinosinusitis, we wanted to investigate the clinical features and explore if the presence of biofilm, nasal polyps or other disease characteristic could serve as predictor for the symptomatic load. A patient group undergoing septoplasty without disease of the sinuses was included as control.

**Methods:**

The Sinonasal outcome test (SNOT-20), EPOS visual analogue scale (VAS) and the Lund-Mackey CT score (LM CT score) were used to examine 23 patients with chronic rhinosinusitis without nasal polyps (CRSsNP), 30 patient with nasal polyps (CRSwNP) and 22 patients with septal deviation. Tissue samples were collected prospectively during surgery. The cohort has previously been examined for the presence of biofilm.

**Results:**

Patients with CRSsNP and CRSwNP had significantly higher degree of symptoms compared to the septoplasty group (SNOT-20 scores of 39.8, 43.6 and 29.9, respectively, *p* = 0.034). There were no significant differences in the total SNOT-20 or VAS symptoms scores between the CRSsNP and CRSwNP subgroups. However patients with nasal polyps showed significantly higher scores of symptoms related to sinonasal discomfort such as cough, runny nose and need to blow nose (*p* = 0.011, *p* = 0.046, *p* = 0.001 respectively). Patients with nasal polyps showed a significantly higher LM CT score compared to patients without polyps (12.06 versus 8.00, *p* = 0.001). The presence of biofilm did not impact the degree of symptoms.

**Conclusion:**

The presence of nasal polyp formations in CRS patients was associated with a higher symptomatic airway load as compared to patients without polyps. These findings suggest that nasal polyps could be an indicator of more substantial sinonasal disease. The presence of biofilm did not impact the degree of symptoms, however, as biofilm seem to be a common feature of chronic rhinosinusitis (89% in this cohort), it is more likely to be involved in the development of the CRS, rather than being a surrogate marker for increased symptomatic load.

**Electronic supplementary material:**

The online version of this article (10.1186/s12901-017-0048-6) contains supplementary material, which is available to authorized users.

## Background

Chronic rhinosinusitis (CRS) is a major contributor to disease in central Europe with prevalence of 10.9% [1]. CRS patients with or without nasal polyps are a heterogeneous group with various phenotypes and characteristics such as allergic rhinitis, eosinophilic inflammation, Samters triad, fungal allergy, *Staphylococcus aureus* superantigens, biofilm formation and some patients without any apparent predispositions. They contribute to a whole range of different clinical setting ranging from severe life threatening disease as intracranial and intraorbital infections, to mild or low-grade chronic inflammation with various degrees of morbidity.

Eradication of chronic rhinosinusitis with medical treatment or surgery can be difficult, one reason for this might be the formation of biofilm in the sinonasal cavities. Bacteria are found in two different states. One state is characterized by free-floating bacteria with rapid bacterial growth, the planktonic state. This represents the traditional understanding of bacterial existence. In the other state, called the biofilm state, the bacteria are organized in a microbial community, often surrounded by an extracellular polymeric matrix. Within the biofilms the bacteria communicate with each other in a process known as quorum sensing. This communication is facilitated by several classes of molecules, amongst them peptides. Quorum sensing can influence a wide range of bacterial actions by regulating genetic expression. One example of this is switching back to the planktonic state. Bacteria in a biofilm can also exchange genetic material, especially genes involved in antibiotic resistance. Biofilms are usually heterogenic, consisting of many different strains of bacteria and/or fungi.

The precise distribution of biofilm in the CRS population has been debated, but there seems to be clear evidence of increased biofilm formation in the CRS patient ranging from 15 to 100% [2–4]. As reported earlier by this group, CRSwNP express a higher prevalence of biofilm as compared to CRSsNP. Biofilm was also found in 56% of the control patients without CRS but with septal deviations. Thus, the definite role of biofilms in CRS is still unclear.

One of the most obvious differences in the CRS groups is the presence or absence of nasal polyps. Previous European studies have shown that 77% of nasal polyp patients are characterized by an eosinophilic inflammation mediated by T helper 2 cells [5]. The reasons for this T-cell activation is still unknown, most likely both extrinsic and intrinsic factors are involved. Studies have indicated that nasal polyps may impact the degree of symptoms and the effect of surgery [6]. The eosinophilic component in CRSwNP, is also present in patients with bronchial eosinophilic hypersensitivity, and there are reports indicating a correlation between asthma and rhinitis [7]. An association between CRS and asthma has been shown, but it is still unknown if patients with CRSwNP are more predisposed to develop asthma when compared to patients with CSRsNP. However, patients with CRS are at higher risk to develop symptoms from the respiratory tract such as bronchial asthma, which have led to the concept of united airway disease.

The goal of this study was to investigate the clinical features and objective findings in a Norwegian CRS cohort previously examined for the prevalence of biofilm [8], and explore if biofilm, nasal polyps or other disease characteristic could serve as predictor for the symptomatic load in such patients.

## Methods

Eighty-seven patients undergoing primary nasal surgery were included from 2010 to 2012 at Akershus University Hospital. 62 patients were diagnosed with CRS according to EPOS (2007) and underwent FESS. 27 of these patient were without polyps, 35 patients had accompanying polyps. 25 patients with nasal stenosis due to septal deviation were included as control, these patients underwent septoplasty. Of the 87 patients included in the study 12 patients (4 CRSsNP, 5 CRSwNP and 3 septoplasty patients) had not fully completed the questionnaires and thus were omitted from the statistical analysis. The patient demographics of this cohort is described in detail in a previous report by this group [2]. The study was approved by the hospital board and the Regional Ethical Committee (reference number 2009/1720b). Written consent was obtained from all patients, the study was performed according to the Helsinki declaration.

### Data collection

Medical history including the duration of CRS, previous medical treatments (nasal- and/or systemic steroids), smoking-history, asthma and allergy were obtained. All patients underwent preoperative Computer Tomography (CT), and were subsequently scored according to Lund Mackay CT scoring system. CRS patients were asked to grade their quality of life and severity of disease using the EPOS 2007 visual analogue score (VAS) and the SNOT-20 questionnaire.

The data were analysed using SPSS 23 IBM. The results are reported as mean values. One-way analysis of variance, ANOVA, was used to compare biofilm formation, LM CT score, SNOT-20 and its subscore between the CRSsNP, CRSwNP and the septoplasty patients. Student’s t-test was used to assess the significance of the mean differences for time to referral, VAS, LM CT score, SNOT-20 and its subscore between CRSsNP and CRSwNP. Pearson correlation was used to evaluate the correlation between Lund Mackey CT score and SNOT-20/VAS, and correlation between SNOT-20 and VAS.

## Results

**Fig. 1:**
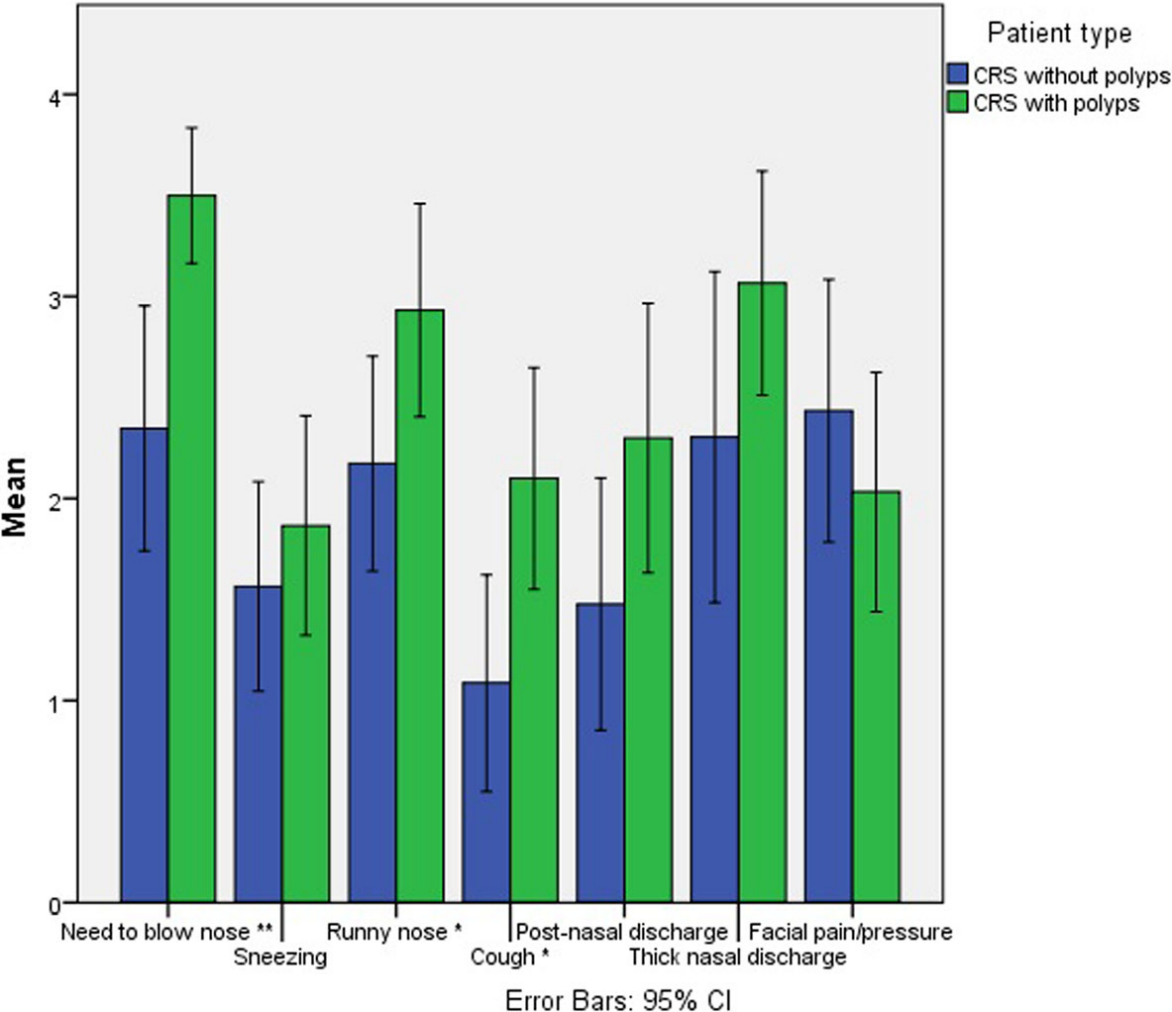
Clinical differences between CRSsNP and CRSwNP

Patients with CRSsNP and CRSwNP had significantly higher degree of symptoms compared to the septoplasty group (SNOT-20: 39.8, 43.6 and 29.9, respectively, ANOVA *p* = 0.03). There were no differences in the total SNOT-20 or VAS scores between the CRSsNP and CRSwNP groups (39.8 vs. 43.6, t-test *p* = 0.455, and 63 vs. 68.6, t-test *p* = 0.265, respectively). Regarding the SNOT questions related to nasal discomfort, patients with nasal polyps showed significantly higher scores as compared to patients without nasal polyps with regards to cough, runny nose and need to blow nose (t-test *p* = 0.011, *p* = 0.046 and *p* = 0.001, respectively). The association of nasal polyps and cough (*p* = 0.011) and need to blow nose (*p* = 0.001), remained statistical significant also when controlling for the presence of allergy as a potential confounder. The statistical significance for the third parameter, runny nose (*p* = 0.046), became insignificant when controlling for allergy in the cohort (*p* = 0.248). No statistical association was found between allergy and this symptomatic parameter (*p* = 0.053). The results are summarized in Table 1 and Fig. 1.

**Table 1:** Mean VAS and SNOT scores for the different patient categories

	Septoplasty	CRSsNP	CRSwNP	*P*-value (ANOVA)	*P*-value (t-test)
Number of patients	22	23	30		
Time to referral (months)		99	59		0.325
VAS score		63	68.6		0.265
SNOT-20 (total score)	29.9	39.8	43.6	0.034	0.455
SNOT (subscore)					
Need to blow nose	2.23	2.35	3.5	<0.001	0.001
Sneezing	1.27	1.57	1.87	0.29	0.425
Runny nose	1.86	2.17	2.93	0.02	0.046
Cough	1.27	1.09	2.10	0.02	0.011
Post-nasal discharge	1.73	1.48	2.3	0.19	0.077
Thick nasal discharge	1.73	2.3	3.07	0.02	0.119
Facial pain/pressure	1.27	2.43	2.03	0.04	0.355

One-way ANOVA was applied to calculate the significance of differences between CRSsNP, CRSwNP and septoplasty patients. The student’s t-test was used to identify differences between CRSsNP and CRSwNP

The mean duration of CRS symptoms prior to evaluation at our clinic was 79 months. The CRSsNP had a mean duration of CRS symptoms of 99 months prior to evaluation, while the CRSwNP had an average duration of 59 months. This difference did not reach statistical significance (t-test *p* = 0.325). The mean Lund Mackay CT score for the CRS group was 10,29. The CRSwNP patients had a higher LM CT score compared to the CRSsNP patients (12.06 vs. 8.00, t-test *p* = 001).

Biofilm formation was found in 81% of the patients with chronic rhinosinusitis without polyps, in 97% of the patient with polyps, and in 56% of the patients undergoing septoplasty as reported previously [2].

There was a significant correlation between the VAS- and SNOT-20 scores Pearson’s correlation coefficient of 0.525 (*p* = 0.001).

## Discussion

In this study we looked at differences in a patient population undergoing primary nasal surgery at Akershus University Hospital (Ahus). Ahus is a regional centre for otorhinolaryngology with patients referred from general practitioners and consultants. We examined the clinical differences between patients operated for CRS with or without nasal polyps and patients operated for septal deviation.

Nasal polyps increase the mass in the nose and mucopurulent stagnation thus as expected CRSwNP had significantly higher LM CT score compared to CRSsNP (12,06 vs. 8,00, t-test *p* = 0,001). As identified by other groups [9] we found a low correlation between the LM CT scores of the CRS patients and the SNOT-20 score (Pearson’s correlation *r* = −0.115). The same was seen for the VAS and Lund Mackay CT scores (Pearson’s correlation *r* = −0.005). Further analysing the subset of SNOT-20 symptoms in Table 1 only the “need to blow nose” subcategory showed a significant correlation to the Lund Mackay CT score (*r* = 0.325, *p* = 0.018). Subdividing the CRS groups into CRSwNP and CRSsNP did not revile any statistical significant correlation between LM CT score and SNOT or VAS values.

One reason for the low correlation between the LM CT score and SNOT-20 scores could be the slow and gradual development of disease, that makes a true baseline difficult to perceive for the patients.

**Fig. 2:**
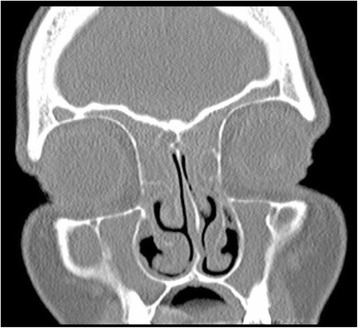
CT picture of patient illustrating an individual variability between CT score and SNOT-20 score

The low correlation between SNOT-20 and Lund Mackay CT score is well illustrated by one of the included patients with a high Lund Mackay CT score of 20 and a relatively low SNOT-20 score of 16 and VAS 52 (Fig. 2).

Previous studies have shown that biofilm formations give rise to worse postoperative outcome [10]. There are only a few investigations into preoperative clinical differences between CRS patient with of without biofilm formation. Li et al. [11] found increase SNOT-20 symptom score and increased nasal- and sinus-related symptoms including need to blow nose, cough and postnasal discharge in their biofilm groups compared to the patients without biofilm. Due to the large prevalence of biofilm among our patients (CRSsNP 81,5%, CRSwNP 97,15% and control patients 56%) we were not able to investigate further any difference in clinical features between the biofilm positive and negative groups.

We have earlier shown [8], that CRSwNP and biofilm formation seems to be closely related. In populations with biofilm formation it is thus expected that the degree of polyp formation and symptomatic load is increased compared to patients without polyps and biofilm.

Even though we found increase SNOT-20 and VAS score in the patients with CRSwNP compared to the CRSsNP patients the differences were not significant. However, when looking at the nose related individual SNOT-20 questions, we found a significant increased score in the polyp patients regarding need to blow nose (2,35 vs. 3,5 t-test *p* = 0,001), runny nose (2,17 vs. 2,93 t-test *p* = 0,046) and cough (1,09 vs. 2,1 t-test *p* = 0,011). As allergic rhinits also cause nose related symptoms we re-analyzed the association of nasal symptoms and polyps while controlling for the presence of allergy. The association of nasal polyps and cough (*p* = 0.011) and need to blow nose (*p* = 0.001), remained statistical significant also when controlling for the presence of allergy as a potential confounder. The statistical significance for the third parameter, runny nose (*p* = 0.046), became insignificant when controlling for allergy in the cohort (*p* = 0.248). To further understand if allergy alone was associated with an increased runny nose, we examined the association between allergy and runny nose directly and found no statistical association between allergy and this symptomatic parameter (*p* = 0.053). Our interpretation of these supplementary analysis is that nasal polyps is associated with increased nasal symptoms, however for runny nose the relationship between nasal polyps, runny nose and allergy is not completely revealed as allergy does impact the statistical significance of nasal polyps driving such symptoms. As allergy alone is not associated with runny nose, it strengthens the impact of nasal polyps on this specific symptomatic finding. Further investigations are necessary to better understand the complete clinical presentation of such patients and underlying biological phenomena.

Nasal discharge, postnasal drip and cough are also symptoms often seen in patients with asthma. There is evidence of a link between asthma and CRS giving rise to the hypothesis of united airways. The exact molecular pathways are still unclear [12] but specially the eosinophilic nasal polyp inflammations share similarities to eosinophilic asthma with a Th-2 inflammatory response pathway. In our population we found a higher number of patients with asthma in the CRS groups (33,3%) compared to the patients with septum deviation (8.7%). We did not find any significant difference in the prevalence of asthma between the CRSwNP and CRSsNP as other investigators have reported [13, 14]. Håkansson et al. 2014 [15] found a 25% under diagnosis of asthma in the CRS group. This may explain the reason for the low number of asthma patients in our cohort as we did not test our patients for asthma but based the diagnosis on medical history and use of asthma medication. Further investigations into these connections are needed.

**Fig. 3:**
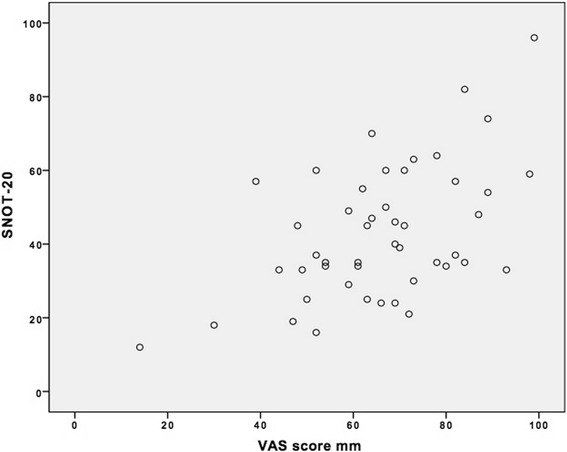
Scatterplot illustrating the correlation between VAS and SNOT-20 score

The VAS score from the EPOS document is a fast and easy tool for assessing severity of sinus symptoms. In or study we found a Pearson’s correlation coefficient of 0.525 (*p* = 0.001) between the VAS score and the SNOT-20 score for our CRS patients (Fig. 3). In a busy practice pressed for time the VAS score gives valuable information regarding patient sinus disease.

## Conclusion

Chronic rhinosinusitis with nasal polyps gives an increased symptomatic load compared to chronic rhinosinusitis without nasal polyps. This is specially seen in relations to symptoms of the united airways as cough, need to blow nose and postnasal discharge. The finding of nasal polyps should therefore alert the physician to expect increased sinonasal discomfort and thus help in the prioritization of medical and surgical treatment. Due to the high prevalence of biofilm formation we were not able to find specific clinical features related to biofilm formation.

## Additional file

Additional file 1: Data containing patient disease classification, allergy status, asthma status, LM CT score, VAS score, SNOT score and biofilm status. (XLS 28 kb)

## Abbreviations

CRS: Chronic rhinosinusitis
CRSsNP: Chronic rhinosinusitis without nasal polyps
CRSwNP: Chronic rhinosinusitis with nasal polyps
LM CT score: Lund Mackay CT score
NP: Nasal polyps
SNOT-20: Sino-Nasal-Outcome-Test-20
VAS: EPOS Visual analogue scale
vs: Versus
